# Correlation between overactive bladder symptom score and neuropsychological parameters in Alzheimer’s disease patients with lower urinary tract symptom

**DOI:** 10.1590/S1677-5538.IBJU.2015.0664

**Published:** 2017

**Authors:** Ha Bum Jung, Don Kyoung Choi, Seong Ho Lee, Sung Tae Cho, Hae Ri Na, Moon Ho Park

**Affiliations:** 1 Department of Urology, College of Medicine, Hallym University, Seoul, Korea Seoul, Korea;; 2 Department of Neurology, Bobath Memorial Hospital, Seongnam-si, Korea;; 3 Department of Neurology, College of Medicine, Korea University, Seoul, Korea

**Keywords:** Neuropsychological Tests, Urinary Bladder, Lower Urinary Tract Symptoms

## Abstract

**Purpose:**

To examine an association between the overactive bladder symptom score (OABSS) and neuropsychological parameters. Moreover, we investigate the factors that affect each item in the questionnaire.

**Materials and Methods:**

A total of 376 patients (males: 184; females: 192) with probable Alzheimer’s disease (AD) were recruited. Cognitive testing was conducted using the Mini Mental Status Examination (MMSE), Clinical Dementia Rating (CDR) scale, Global Deterioration Scale (GDS), and Barthel Activities of Daily Living (ADL). Lower urinary tract symptom (LUTS) was assessed using OABSS and voiding diary.

**Results:**

The prevalence of overactive bladder (OAB) (defined as OABSS ≥3 with an urgency score of ≥2) in patients with AD was 72.6%. Among the OAB subjects, the most common severity of symptom was moderate (72.6%), followed by mild (21.2%), and severe (5.8%). It was found that OABSS had a very high correlation with aging (r=0.75; p<0.001). When compared with neuropsychological parameters, it was found that OABSS was highly correlated with the CDR scores (r=0.446; p<0.001). However, no significant correlation was found between the changes in OABSS scores and those in other neuropsychological parameters. Based on the individual symptom scores, urgency incontinence was highly correlated with the CDR scores (r=0.43; p<0.001).

**Conclusions:**

OABSS is a useful tool in assessing AD patients with LUTS. There was a consistent positive association between OABSS severity, including urgency incontinence, and CDR scores.

## INTRODUCTION

Overactive bladder (OAB) is a symptom complex, comprising urinary urgency with or without urgency and incontinence, usually with urinary frequency and nocturia (1); it represents the storage component of lower urinary tract symptoms (LUTS) (2). The incidence of OAB increases significantly with age. The mechanisms underlying OAB in the elderly are multifactorial; the factors might include age-related changes in the bladder itself, or central nervous system changes innervating the bladder (3).

Alzheimer’s disease (AD) is one of the most common neurodegenerative diseases and accounts for more than 80% of dementia patients among elderly people (4). The condition is associated with progressive memory loss, and impairment of cognitive function and functional independence. Many affected patients will also have problems with bladder and bowel control (5, 6). Both OAB and AD are common, often coexisting in older patients (7). Although AD is known to be an independent risk factor for OAB or urinary incontinence, few studies have been published with regard to research on OAB of elderly patients with AD (3, 8).

As for the relationship between urinary incontinence and cognitive or functional measures in AD patients, we previously reported that severity of detrusor overactivity was linked to functional impairments, whereas there was no relationship between the Incontinence Questionnaire on Urinary Incontinence Short Form (ICIQ-UI) questionnaire and those (5). However, there has been a lack of study to investigate in detail the relationship between OAB with each symptom and cognitive or functional measures in AD patients with LUTS. Therefore, we assessed the OAB of patients with AD, examined the association between the Overactive Bladder Symptom Score (OABSS) questionnaire and neuropsychological parameters, and investigated the factors that affect each item of the questionnaire.

## MATERIALS AND METHODS

Subjects who visited the dementia clinic were recruited sequentially. All patients met the National Institute of Neurological Communicative Disorders and Stroke (NINCDS) and the Alzheimer Disease and Related Disorders Association diagnostic criteria for probable AD. The diagnosis of probable AD was made by expert neurologists. Patients who were diagnosed with other dementia, including severe dementia in addition to behavioral disturbances, and inability to communicate were also excluded.

All participants underwent an extensive evaluation that included physical and neurological examinations and laboratory test. Cognitive tests were performed using the Mini Mental Status Examination (MMSE), Clinical Dementia Rating (CDR) scale, Global Deterioration Scale (GDS), and Barthel Activities of Daily Living (ADL), which have extensively been used in clinical and research settings to measure cognitive impairment. LUTS were assessed using the OABSS questionnaire and 3-day consecutive voiding diary with the Indevus Urgency Severity Scale (IUSS). OAB was defined as OABSS ≥3 with an urgency score of ≥2 (9, 10). In addition, scores on the OABSS of ≤5 were defined as mild, those of 6-11 as moderate, and those of ≥12 as severe (9, 10).

The institutional review board approved the study protocol, and informed consent was obtained from all patients or legal guardians in accordance with the Declaration of Helsinki. Patients were asked to complete by themselves a questionnaire and voiding diary. However, if they were unable to complete them due to behavioral disturbances and inabilities, educated caregivers asked them the questions from the questionnaire and assisted them in filling out the required forms.

Data are expressed as means and standard deviations. A p-value was calculated using the independent t-test, Pearson’s chi-squared test, and one-way analysis of variance. A Pearson correlation analysis was used to determine the correlations between the individual symptom scores of OABSS and neuropsychological parameters. All tests with a p-value of <0.05 were considered as statistically significant. The Statistical Package for the Social Sciences Version 18.0 (SPSS, Chicago, IL) was used to carry out all statistical analyses.

## RESULTS

A total of 376 patients (male 184, female 192, 56-92 years old), with probable AD, were included in the analysis. Of 430 patients screened, 54 were not included because of diagnosis of other dementia, any severe conditions of behavioral disturbances, and inability to communicate. The prevalence of OAB (defined as OABSS ≥3 with an urgency score of ≥2) in patients with AD was 72.6% (n=273; males: 42.1%; females: 57.9%). Of those, 260 patients (95.2%) complained of urinary leakage associated with urgency (urgency incontinence score of ≥1) and 90 patients (33.0%) were incontinent more than once per day (urgency incontinence score of ≥3). However, only 56 patients (20.5%) used adult diapers or pads for incontinence or night wetting. Table-1 summarizes the variable parameters of patients with and without OAB. No statistical differences among age, sex, duration of disease, history of taking acetylcholinesterase inhibitors (AChEIs), and neuropsychological parameters were found between those with and without OAB.

**Table 1:** The prevalence of OAB on OABSS (n=376).

	OAB (n=273)	Non-OAB (n=103)	P-value
Age (years)	78.21±7.70	77.47±8.65	0.418
(range)	58–92	56–91	
Sex (M/F)	115/158	39/64	0.454
Hypertension	103 (37.7%)	43 (41.7%)	0.476
Diabetes	75 (27.4%)	22 (21.4%)	0.227
Dyslipidemia	91 (33.3%)	31 (30.1%)	0.550
**Duration**			
Of education (y)	6.57±4.36	7.14±5.02	0.693
Of dementia (m)	33.73±16.39	28.84±19.99	0.540
History of taking AChEIs (%)	115 (42.1%)	43 (41.7%)	0.947
MMSE	14.44±7.62	14.92±7.78	0.589
CDR	2.27 ± 0.97	2.21 ± 0.97	0.643
GDS	5.54 ± 0.98	5.57 ± 0.95	0.250
ADL	12.60±6.46	12.10±6.12	0.488

Values are presented as mean ± standard deviation.

**OAB** = overactive bladder; **AChEIs** = acetylcholinesterase inhibitors; **MMSE** = the Mini Mental Status Examination; **CDR** = the Clinical Dementia Rating scale; **GDS** = the Global Deterioration Scale; **ADL** = the Barthel Activities of Daily Living.

When the severity of symptom was categorized as mild (OABSS: ≤5), moderate (OABSS: 6–11), and severe (OABSS: ≥12), the most common was moderate (72.6%), followed by mild (21.2%), and severe (5.8%). The mean age of patients with mild, moderate, and severe symptoms of OABSS was 70.8, 79.3, and 91.3, respectively. Urgency episodes and maximum urgency intensity were significantly increased in the severe group. However, there were no significant differences in the number of micturition, nocturia and mean voided volume among the groups. Table-2 summarizes the parameters of OAB patients that were classified into three groups in accordance with the severity of OAB. Among the neuropsychological tests, only CDR increased significantly with the severity of OAB. The MMSE, GDS, and ADL did not significantly differ among the three groups.

**Table 2:** The severity of OAB on OABSS (n = 273).

	Mild	Moderate	Severe	P-value

	(OABSS ≤5)	(OABSS 6–11)	(OABSS ≥12)	
N (%)	58 (21.2%)	199 (72.6%)	16 (5.8%)	
Age (years)	70.8±6.2	79.3±6.2	91.3±0.79	<0.001
Sex (M/F)	30/28	81/118	4/12	0.118
**Voiding diary (/24 hr)**				
No. micturition	8.7±2.7	9.1±2.9	10.3±3.3	0.147
No. nocturia	1.2±0.8	1.2±0.9	1.6±1.0	0.144
No. urgency episodes	1.2±0.9	1.2±0.9	2.4±0.8	<0.001
Max. urgency intensity	1.6±0.9	1.6±0.9	2.2±0.9	0.038
Mean voided volume (mL)	127.3±45.7	122.9±41.7	118.1±28.0	0.675
**Neuropsychological tests**				
MMSE	14.36±7.31	14.59±7.74	12.94±7.51	0.705
CDR	1.76±0.79	2.32±0.94	3.38±0.72	<0.001
GDS	5.57±0.86	5.38±1.01	5.75±1.00	0.195
ADL	12.02±5.18	12.66±6.83	14.13±5.95	0.504

Values are presented as mean ± standard deviation.

**MMSE** = the Mini Mental Status Examination; **CDR** = the Clinical Dementia Rating scale; **GDS** = the Global Deterioration Scale; **ADL** = the Barthel Activities of Daily Living.

In the correlation analysis, OABSS had a very strong relationship with aging in AD patients with OAB (r=0.75; p<0.001). When compared with neuropsychological parameters, OABSS highly correlated with the CDR scores in AD patients with OAB. However, no significant correlation was found between changes in OABSS and the other three neuropsychological tests-MMSE, GDS, and ADL (Table-3).

**Table 3:** Correlations between neuropsychological parameters and OABSS scores.

	Pearson correlation with OABSS scores	P-value
MMSE	−0.027	0.65
CDR	0.446	<0.001
GDS	0.006	0.93
ADL	0.048	0.43

**MMSE** = the Mini Mental Status Examination; **CDR** = the Clinical Dementia Rating scale; **GDS** = the Global Deterioration Scale; **ADL** = the Barthel Activities of Daily Living.

Based on individual symptom scores, urgency incontinence highly correlated with the CDR scores (r=0.43; p<0.001). However, the frequency and nocturia scores indicated weak correlation (r=0.22 and 0.23). There was no correlation between individual symptom scores and other neuropsychological parameters (Table-4).

**Table 4:** The individual symptom scores of OABSS and correlations with neuropsychological parameters.

	Frequency	Nocturia	Urgency	Urgency Incontinence
MMSE	−0.16	−0.09	−0.03	0.03
CDR	0.22*	0.23*	0.11	0.43*
GDS	0.06	−0.07	−0.02	0.03
ADL	−0.02	0.05	0.11	−0.01

* P < 0.001

**MMSE** = the Mini Mental Status Examination; **CDR** = the Clinical Dementia Rating scale; **GDS** = the Global Deterioration Scale; **ADL** = the Barthel Activities of Daily Living.

## DISCUSSION

OAB is a troublesome and extremely prevalent urinary symptom that causes a significant negative impact on the quality of life (QoL), associated with high economic costs or related comorbidities, particularly in elderly people. Many studies have shown that OAB can affect all aspects of QoL, including psychological, physical, sexual, domestic, social, and occupational aspects (11).

The incidence of OAB increases with aging, and central nervous system (CNS) degeneration in the elderly is proposed as one of the pathogenic factors of OAB (12). In our study, the overall prevalence of OAB between AD patients aged 56-92 was 72.6%, which is much higher than those of the general population, as reported in previous studies (13, 14) Furthermore, it is nearly twice those of the general population aged ≥75 in the studies. From the aspect of the severity of OAB symptom, elderly AD patients with OAB tend to have greater severe symptoms (r=0.75; p<0.001). However, there was no significant difference of age between AD patients with and without OAB.

We also analyzed the score of MMSE, CDR, GDS, and ADL of subjects to determine the relationship between OAB symptoms and the severity of AD. AD is diagnosed based on medical history, family history, and general behavioral observations. Diagnosis criteria require the presence of cognitive impairment and suspected dementia symptoms. It was confirmed through neuropsychological tests, such as MMSE, CDR, GDS, and ADL, which have shown good statistical reliability and validity compared with the definitive histopathological confirmation of brain tissues (15, 16).

MMSE is a 30-point questionnaire that is used in clinical and research settings to measure cognitive impairment (17). This test takes between 5–10 minutes and examines the following: registration, attention and calculation, recall, language, ability to follow simple commands and orientation (18). A previous study reported a correlation between nocturia and MMSE score, with a lower MMSE score being nocturia (17, 19). However, in our study, there was no significant difference of MMSE scores between AD patients with and without OAB. In addition, MMSE did not significantly differ with the severity of OAB or individual symptom scores, including nocturia. While the advantage of MMSE is its short administration period and ease of use, its disadvantage is that it is affected by educational level, age, and insensitivity to progressive changes (20).

CDR is a numeric scale used to quantify the severity of dementia symptoms. This scale assesses patient’s cognitive and functional performance in six areas: memory, orientation, judgment and problem solving, community affairs, home and hobbies, and personal care. CDR could identify very mild impairments, but its drawbacks include its length of administration, ultimate reliance on subjective assessment, and relative inability to capture changes over time (21). We found a strong correlation between OABSS and CDR scores (Table-3). In addition, among OABSS scores, we also found a strong correlation between urgency incontinence scale and CDR scores (Table-4). It was well documented that among OAB symptoms, the urgency incontinence was the most frequent symptom in patients with AD (5, 22). Our study showed similar results with that of previous studies. CDR is being used as an effective tool for staging the severity of dementia, while MMSE scores are influenced by age, sex, and educational levels. Therefore, a correlation between OAB and CDR might be significant by itself. After all, our results suggest that patients with greater severity of AD also tend to have greater severity of OAB, including urgency incontinence.

GDS developed by Dr. Barry Reisberg provides caregivers an overview of the stages of cognitive function for those suffering from a primary degenerative dementia such as AD (23). It is subdivided into 7 different stages. Stages 1-3 are the pre-dementia stages. Stages 4-7 are the dementia stages. Beginning in stage 5, an individual can no longer survive without assistance (23). Clinicians broadly accept the GDS as a tool for staging severity of dementia. However, unlike CDR, GDS was not associated with OAB in our study. It is because not all patients with AD follow the course described in the GDS (24).

We also used Barthel ADL to measure basic ADL. Barthel ADL was the scale of functional limitation which mainly evaluated the basic and physical activities of daily living, including dressing, using the toilet, getting about the house, getting in and out of bed, and bathing (22). Instrumental ADL (IADL) was the other scale of functional limitation, which mainly evaluated the more complex and higher-order skills (22). A previous study reported an association between urinary incontinence and IADL score in elderly women (25). However, although the basic ADL had more fundamental items, we could not find an association between OABSS and basic ADL in our study. This may be the case because we excluded very advanced severe dementia.

As far as we know, a few studies have been conducted in evaluating OAB of AD patients through a standardized questionnaire. This is possibly due to the difficulty of AD patients in completing a questionnaire or doubts about the inexactitude of their responses. Nevertheless, an assessment of symptoms is essential in clinical OAB diagnosis or treatment, and it is not exceptional in OAB management of AD patients (26, 27). Symptom questionnaires overcome historical drawbacks, such as missed questions, being led by questioners, or quantification difficulty because of the structured panel of questions in diagnosing OAB. OABSS was first introduced in 2006 and currently, it is one of the most widely used questionnaires for OAB treatment or research (10, 28). Among its advantages are exact compatibility with the ICS definition of OAB, as well as its simplicity and brevity. OABSS, which consists of only four questions, is nearly the simplest questionnaire available (10, 29-31). Thus, we thought that OABSS is very suitable in the evaluation of AD patients because many of them have cognitive impairment. Moreover, its simplicity can help in the reduction of confusion and in drawing exact responses as much as possible.

LUTS is a group of symptoms (voiding and storage) that is supposed to be expressed by the patients themselves. Thus, studying LUTS in patients with brain diseases is not easy as they are older or have cognitive disorders, which make them unable to fully cooperate. A standardized questionnaire could be helpful, and we tried to evaluate the OAB of AD patients using OABSS while remaining faithful to the definition of OAB. Still, we cannot be convinced that AD patients genuinely expressed OAB symptoms exactly as with the general population, even though we excluded patients with severe cognitive disorders. The result where the other three neuropsychological scores were not correlated with OABSS is another shortcoming of this study. Our limitations need to be solved through more novel research designs in the future.

## CONCLUSIONS

The prevalence of OAB in AD patients is much higher than that of the general population. AD patients with OAB have more severe OAB symptoms as they age. Patients with more severe AD, based on CDR, tend to have more severe OAB symptoms. Thus, results of the present study suggest that OABSS could be a useful tool in the assessment of OAB symptoms of AD patients.

## References

[1] Abrams P, Cardozo L, Fall M, Griffiths D, Rosier P, Ulmsten U (2002). The standardisation of terminology of lower urinary tract function: report from the Standardisation Sub-committee of the International Continence Society. Am J Obstet Gynecol.

[2] Wagg AS, Cardozo L, Chapple C, De Ridder D, Kelleher C, Kirby M (2007). Overactive bladder syndrome in older people. BJU Int.

[3] Sakakibara R, Panicker J, Fowler CJ, Tateno F, Kishi M, Tsuyusaki Y (2014). Is overactive bladder a brain disease? The pathophysiological role of cerebral white matter in the elderly. Int J Urol.

[4] Anand R, Gill KD, Mahdi AA (2014). Therapeutics of Alzheimer’s disease: Past, presente and future. Neuropharmacology.

[5] Lee SH, Cho ST, Na HR, Ko SB, Park MH (2014). Urinary incontinence in patients with Alzheimer’s disease: relationship between symptom status and urodynamic diagnoses. Int J Urol.

[6] Griebling TL (2015). Re: Urinary incontinence in patients with Alzheimer’s disease: relationship between symptom status and urodynamic diagnoses. J Urol.

[7] Ouslander JG (2004). Management of overactive bladder. N Engl J Med.

[8] Takahashi O, Sakakibara R, Panicker J, Fowler CJ, Tateno F, Kishi M (2012). White matter lesions or Alzheimer’s disease: which contributes more to overactive bladder and incontinence in elderly adults with dementia?. J Am Geriatr Soc.

[9] Ohgaki K, Horiuchi K, Kondo Y (2012). Association between metabolic syndrome and male overactive bladder in a Japanese population based on three different sets of criteria for metabolic syndrome and the Overactive Bladder Symptom Score. Urology.

[10] Homma Y, Yoshida M, Seki N, Yokoyama O, Kakizaki H, Gotoh M (2006). Symptom assessment tool for overactive bladder syndrome--overactive bladder symptom score. Urology.

[11] Brown JS, McGhan WF, Chokroverty S (2000). Comorbidities associated with overactive bladder. Am J Manag Care.

[12] Tadic SD, Griffiths D, Schaefer W, Murrin A, Clarkson B, Resnick NM (2012). Brain activity underlying impaired continence control in older women with overactive bladder. Neurourol Urodyn.

[13] Milsom I, Abrams P, Cardozo L, Roberts RG, Thüroff J, Wein AJ (2001). How widespread are the symptoms of an overactive bladder and how are they managed? A population-based prevalence study. BJU Int.

[14] Stewart WF, Van Rooyen JB, Cundiff GW, Abrams P, Herzog AR, Corey R (2003). Prevalence and burden of overactive bladder in the United States. World J Urol.

[15] Dubois B, Feldman HH, Jacova C, Dekosky ST, Barberger-Gateau P, Cummings J (2007). Research criteria for the diagnosis of Alzheimer’s disease: revising the NINCDS-ADRDA criteria. Lancet Neurol.

[16] Blacker D, Albert MS, Bassett SS, Go RC, Harrell LE, Folstein MF (1994). Reliability and validity of NINCDS-ADRDA criteria for Alzheimer’s disease. The National Institute of Mental Health Genetics Initiative. Arch Neurol.

[17] Lee YJ, Jeong SJ, Byun SS, Lee JJ, Han JW, Kim KW (2012). Prevalence and correlates of nocturia in community-dwelling older men: results from the korean longitudinal study on health and aging. Korean J Urol.

[18] Tuijl JP, Scholte EM, de Craen AJ, van der Mast RC (2012). Screening for cognitive impairment in older general hospital patients: comparison of the Six-Item Cognitive Impairment Test with the Mini-Mental State Examination. Int J Geriatr Psychiatry.

[19] Burgio KL, Johnson TM, Goode PS, Markland AD, Richter HE, Roth DL (2010). Prevalence and correlates of nocturia in community-dwelling older adults.. J Am Geriatr Soc.

[20] Harrell LE, Marson D, Chatterjee A, Parrish JA (2000). The Severe Mini-Mental State Examination: a new neuropsychologic instrument for the bedside assessment of severely impaired patients with Alzheimer disease. Alzheimer Dis Assoc Disord.

[21] Lim WS, Chong MS, Sahadevan S (2007). Utility of the clinical dementia rating in Asian populations. Clin Med Res.

[22] Na HR, Park MH, Cho ST, Lee BC, Park S, Kim KH (2015). Urinary incontinence in Alzheimer’s disease is associated with Clinical Dementia Rating-Sum of Boxes and Barthel Activities of Daily Living. Asia Pac Psychiatry.

[23] Reisberg B, Ferris SH, de Leon MJ, Crook T (1982). The Global Deterioration Scale for assessment of primary degenerative dementia. Am J Psychiatry.

[24] Eisdorfer C, Cohen D, Paveza GJ, Ashford JW, Luchins DJ, Gorelick PB (1992). An empirical evaluation of the Global Deterioration Scale for staging Alzheimer’s disease. Am J Psychiatry.

[25] Park J, Hong GR, Yang W (2015). Factors Associated With Self-reported and Medically Diagnosed Urinary Incontinence Among Community-Dwelling Older Women In Korea. Int Neurourol J.

[26] Gormley EA, Lightner DJ, Faraday M, Vasavada SP (2015). Diagnosis and treatment of overactive bladder (non-neurogenic) in adults: AUA/SUFU guideline amendment. J Urol.

[27] Yamaguchi O, Nishizawa O, Takeda M, Yokoyama O, Homma Y, Kakizaki H (2009). Neurogenic Bladder Society. Clinical guidelines for overactive bladder. Int J Urol.

[28] Gotoh M, Homma Y, Yokoyama O, Nishizawa O (2011). Responsiveness and minimal clinically important change in overactive bladder symptom score. Urology.

[29] Avery K, Donovan J, Peters TJ, Shaw C, Gotoh M, Abrams P (2004). ICIQ: a brief and nrobust measure for evaluating the symptoms and impact of urinary incontinence. Neurourol Urodyn.

[30] Coyne K, Revicki D, Hunt T, Corey R, Stewart W, Bentkover J (2002). Psychometric validation of an overactive bladder symptom and health-related quality of life questionnaire: the OAB-q. Qual Life Res.

[31] Barry MJ, Fowler FJ, O’Leary MP, Bruskewitz RC, Holtgrewe HL, Mebust WK (1992). The American Urological Association symptom index for benign prostatic hyperplasia. The Measurement Committee of the American Urological Association. J Urol.

